# Supplementary figures and images for: National mapping of schistosomiasis, soil-transmitted helminthiasis and anaemia in Yemen: Towards better national control and elimination
Source: PLoS Negl Trop Dis. 2022 Mar 25;16(3):e0010092. doi: 10.1371/journal.pntd.0010092 (PMC8986123; doi:10.1371/journal.pntd.0010092)

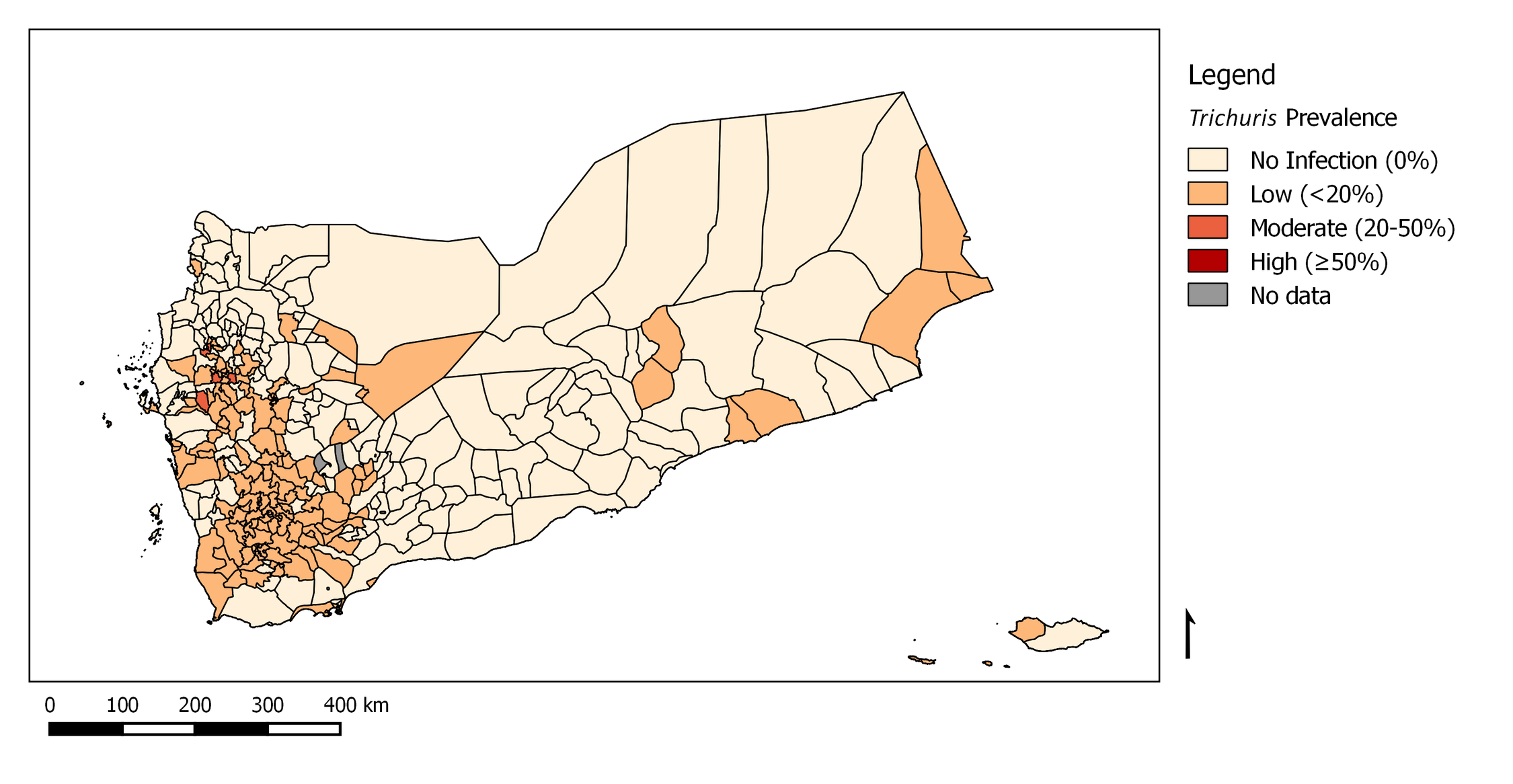

Supplement: S1 Fig — This figure was created for this manuscript in QGIS using open-source data from DIVA-GIS for the base layers. (DIVA-GIS-http://www.diva-gis.org/gdata). (TIF) [file pntd.0010092.s001.tif]

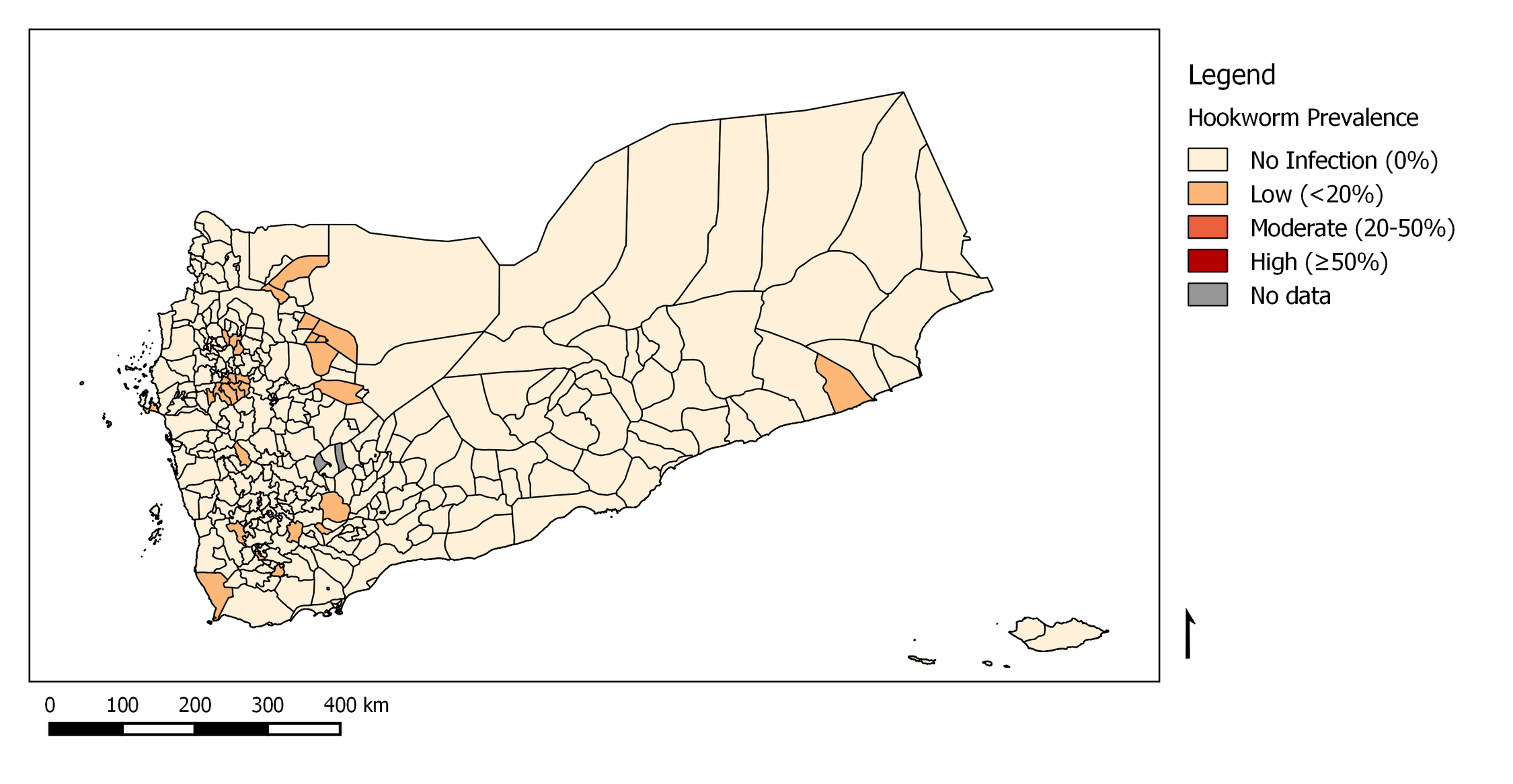

Supplement: S2 Fig — This figure was created for this manuscript in QGIS using open-source data from DIVA-GIS for the base layers. (DIVA-GIS-http://www.diva-gis.org/gdata). (TIF) [file pntd.0010092.s002.tif]
