# Supplementary material for: National mapping of schistosomiasis, soil-transmitted helminthiasis and anaemia in Yemen: Towards better national control and elimination
Source: PLoS Negl Trop Dis. 2022 Mar 25;16(3):e0010092. doi: 10.1371/journal.pntd.0010092 (PMC8986123; doi:10.1371/journal.pntd.0010092)
Supplement: Attachment — Submitted filename: Manuscript Review.docx [file pntd.0010092.s004.docx]

Manuscript Review:

National Mapping of schistosomiasis, soil-transmitted helminthasis and anemia in Yemen: towards better national control and elimination

This manuscript presents the findings of a large nationwide survey for schistosomiasis, soil-transmitted helminthiasis and anemia in Yemen conducted in 2014. Their findings indicate that all three conditions are highly prevalent in Yemen, despite wide scale preventative chemotherapy, and further efforts are thus warranted. Although overall, I support the publication of this manuscript there are several points that need to be addressed before it is ready.

Introduction:

Lines 101-102: The authors mention that the their study is the first national survey, which perhaps it is but it would also be worth mentioning the findings from Nagi et al’s survey conducted in two governorates in 1992 to demonstrate that surveys have been carried out. Would also be good to mention any others that have been conducted anywhere in the country to get a better picture of the historical situation related to SCH and STH. I also find it interesting that no nationwide surveillance had been conducted prior to the implementation of a large-scale preventative chemotherapy campaign in 2010. Perhaps the authors could comment on this or at least provide some more information about the control and prevention activities that have been implemented. For example, in line 102 there is mention that SCH control was sporadic until 2008. It would be beneficial to the reader to have more information here.

Methods and results:

Although overall I found the methods to be well described, the main issue I have with the manuscript is that the authors refer to ‘baseline’ and ‘follow up’ but this study is clearly not a pre/post study. From my understanding, this study was a cross-sectional nationwide prevalence survey so it is not clear what the authors are referring to when using the term ‘baseline’. Are they referring to the desk review and patient records from health facilities mentioned in the abstract? Furthermore, the authors have compared their 2014 survey findings to this ‘baseline’ data in the results (figure 3) but this data has not been provided elsewhere in the manuscript. Either the baseline data needs to be clearly presented in the results and described in the methods or all reference to it in the results (and comparison findings in the discussion) needs to be removed. As an alternative, the authors can refer to this historical data and how it relates to the 2014 survey results in the discussion.

Maps: I found it very difficult to read the maps properly with the names overlaid even when I enlarged the figures. Perhaps these could be improved to make it easier to see the overall pattern in prevalence across districts.

Lines 257-258: can the authors explain how they determined the value for the general population? I assume this is extrapolated from the population of these districts or villages but it is not clear.

Lines 269-270: the authors state 1% in parentheses for *S. haematobium* but according the Table 4 it should be 0.3%.

Lines 294-295, 300-302 and 317-318: these sentences should be in the discussion not results.

Table 4: cell contents cannot be read properly in the headings

Another comment regarding the results is that there is no data on age and infection. This would be interesting to see or even mention if no differences were observed.

Discussion

Lines 377-379: It would be good if the authors could expand a little on this statement (e.g. provide examples).

Lines 398-402: these challenges were not addressed or mentioned in the results, i.e. how did this impact sample population/response rate?

Minor comments:

- Wording in the abstract (lines 53-54) and in discussion: the authors state that ‘one quarter of Yemen districts had severe or moderate *A. lumbricoides* infections’ but the wording implies intensity of infection. Also, ‘infection rate’ is used (line 71) which most often refers to incidence rate and not prevalence, which is measured in this study. I suggest modifying the wording to make clear that they are referring to prevalence of infection.
- There is inconsistency throughout the manuscript with the use of decimals when referring to prevalence
- Few typographical errors throughout the manuscript that need correcting.
