# Supplementary material for: National mapping of schistosomiasis, soil-transmitted helminthiasis and anaemia in Yemen: Towards better national control and elimination
Source: PLoS Negl Trop Dis. 2022 Mar 25;16(3):e0010092. doi: 10.1371/journal.pntd.0010092 (PMC8986123; doi:10.1371/journal.pntd.0010092)
Supplement: Attachment — Submitted filename: Cover Letter.docx [file pntd.0010092.s005.docx]

**Cover letter in response to reviewers’ comments**

**National Mapping of Schistosomiasis, Soil-Transmitted Helminthiasis and Anaemia in Yemen: Towards Better National Control and Elimination**

**PLOS Neglected Tropical Diseases: PNTD-D-21-00800**

Dear Editor,

Thank you for the opportunity to amend the manuscript and to resubmit it to PLOS NTDs for consideration. We thank the anonymous reviewers for their thoughtful review and constructive comments. We have endeavoured to incorporate these suggestions wherever possible, and we feel that these have resulted in a stronger manuscript that better communicates this important area of public health.

Below we provide individual responses to the reviewers’ comments and suggestions and highlight the relevant line numbers from the manuscript.

Thank you for considering this revised version, we look forward to further comments and hopefully a successful publication with PLOS NTDs.

Dhekra Annuzaili, on behalf of the authors

## Reviewer 1

### Comment 1:

Introduction:

Lines 101-102: The authors mention that their study is the first national survey, which perhaps it is but it would also be worth mentioning the findings from Nagi et al’s survey conducted in two governorates in 1992 to demonstrate that surveys have been carried out.

**Response:**

We have referred to Nagi et al’s survey and added the following paragraph about this survey, see page 5 in the introduction, highlighted in yellow.

“There had been several historical surveys conducted at the sub-national level in Yemen. For example, Nagi and colleagues demonstrated that both urogenital and intestinal schistosomiasis were highly prevalence in Taiz governorate and Khamer district (Nagi M et al., 1999). However, no national survey had been implemented before the one described in this current paper.”

### Comment 2:

Would also be good to mention any others that have been conducted anywhere in the country to get a better picture of the historical situation related to SCH and STH. I also find it interesting that no nationwide surveillance had been conducted prior to the implementation of a large-scale preventative chemotherapy campaign in 2010. Perhaps the authors could comment on this or at least provide some more information about the control and prevention activities that have been implemented. For example, in line 102 there is mention that SCH control was sporadic until 2008. It would be beneficial to the reader to have more information here.

**Response:**

We have added the following paragraph on page 6, highlighted in yellow:

“Up until 2008, treatment against SCH and STH was sporadic, at local scale, and dependent on demand at health facilities and local private pharmacies, in addition to targeting schools in high endemic areas associated with on-the-spot parasitological investigation and treatment. In 2008, a national scale treatment program against SCH and STH was formulated, led by the Yemen Ministry of Public Health and Population (MoPHP), with funding from the World Bank (WB) and The END Fund, and technical support of the Schistosomiasis Control Initiative (SCI) and the WHO [10]. This program treated approximately two million school-aged children in its first year (2008) via preventive chemotherapy using the anthelminthic drug praziquantel (PZQ), to which albendazole (ALB) or mebendazole (MEB) were added for STH. Later, in 2010, the program was expanded to include both schools and communities nationwide. Based on historical records, the program started in 2008 targeting the areas known to be of the highest risk of infection. To gain estimates of SCH prevalence nationwide to inform the treatment approach, in 2010 the program implemented subnational parasitological surveys that sampled students from 108 schools in 27 districts of 9 Yemeni governorates. The analysis utilized a collated database of 41,381 mapped villages as the base for all estimations and extrapolations including village population, number of houses, number of families, male and female population, population under five years old, 6 – 18 years old, and 19 years old and above. The data were combined in a geospatial model with relevant ecological covariates (rivers and other water sources, rainfall, temperature, population distribution, land cover and altitude) to extrapolate across the country with the purpose of stratifying Yemen districts according to level of risk [10].

Comment 3:

Methods and results:

Although overall I found the methods to be well described, the main issue I have with the manuscript is that the authors refer to ‘baseline’ and ‘follow up’ but this study is clearly not a pre/post study. From my understanding, this study was a cross-sectional nationwide prevalence survey so it is not clear what the authors are referring to when using the term ‘baseline’. Are they referring to the desk review and patient records from health facilities mentioned in the abstract? Furthermore, the authors have compared their 2014 survey findings to this ‘baseline’ data in the results (figure 3) but this data has not been provided elsewhere in the manuscript. Either the baseline data needs to be clearly presented in the results and described in the methods or all reference to it in the results (and comparison findings in the discussion) needs to be removed. As an alternative, the authors can refer to this historical data and how it relates to the 2014 survey results in the discussion.

**Response:**

We agree with the reviewer’s point here. The main objective of the paper is to describe the nationwide distribution of SCH and STH infection, and anaemia prevalence, using the results of the 2014 survey. We have made changes to wording throughout the manuscript to make this clearer. As a secondary objective, we do also reference the earlier, baseline, mapping to give an indication of changes in infection distribution. We have provided more information on the design of the earlier mapping in introduction section. We have inserted the following paragraph in page 7, highlighted in yellow, which indicates that the methodology for the two surveys was not identical which precludes a direct comparison. However, we feel presenting the data (with these caveats) is still useful.

“…evaluating the progress, the programme had made against SCH compared to the baseline situation and to potentially shift from an objective of control to elimination. Although we note that the baseline and current surveys used different methodologies meaning a direct comparison was not possible.”

### Comment 4:

Maps: I found it very difficult to read the maps properly with the names overlaid even when I enlarged the figures. Perhaps these could be improved to make it easier to see the overall pattern in prevalence across districts.

**Response:**

We have added higher resolution maps throughout the document to make them easier to read and to aid clarity. The overlaid names in all district-level maps were also removed.

### Comment 5:

Lines 257-258: can the authors explain how they determined the value for the general population? I assume this is extrapolated from the population of these districts or villages but it is not clear.

**Response:** It was not quite clear whether the reviewer was referring to estimates of population or estimates of infection at the population level. If the reviewer’s question is about estimates of population these were based on the population demography of Yemen, as reported by the UN Population Projection, 2014. We have added this information to page 14.

If the question is about estimates of infection at the population level, the WHO guidance is that school-aged children can be surveyed and provide a reasonable proxy of infection at the community level (WHO, 2011). This is the approach that has been used here and outlined in the methods section on page 8, see this reference:

WHO. Helminth control in school-aged children: A guide for managers of control programmes. 2nd ed: Geneva: World Health Organization; 2011.

### Comment 6:

Lines 269-270: the authors state 1% in parentheses for S. haematobium but according the Table 4 it should be 0.3%.

**Response:** Thank you for spotting this mistake. This has been changed.

### Comment 7:

Lines 294-295, 300-302 and 317-318: these sentences should be in the discussion not results.

**Response:** These have been changed.

### Comment 8:

Table 4: cell contents cannot be read properly in the headings

**Response:** The formatting of Table 4 has been updated to make it clearer

### Comment 9:

Another comment regarding the results is that there is no data on age and infection. This would be interesting to see or even mention if no differences were observed.

**Response:** An analysis of age on infection was not included given the narrow range of ages sampled (target of children aged 10-14 years old). In relation to sex, we note on page 8 at the start of the results section:

“Approximately 61% of the samples were male due to poor compliance among female students in various schools.”

Also, in relation to sex, the following has been added to page 15:

“There were no significant sex differences in the prevalence of SCH.”

The following on page 17:

“Infection [of STH] was higher among females than males.”

The following on page 19:

“There was no significant sex difference in the prevalence of anaemia (31.0% and 30.1% among females and males, respectively).”

### Comment 10:

Discussion

Lines 377-379: It would be good if the authors could expand a little on this statement (e.g., provide examples).

**Response:**

We have expanded the discussion on that and provided more details, see page 26

### Comment 11:

Lines 398-402: these challenges were not addressed or mentioned in the results, i.e., how did this impact sample population/response rate?

**Response:**

These challenges discussed here are about the program as a whole. We have mentioned in the study design how unstable security situation influenced sampling; the quotation below has been added as the last sentence in the first paragraph in the study design section, page 8.

“Only one district (Wald Rabi’ district from Al Bayda’ governorate) was excluded in this survey because of its unstable security situation”.

We have added a sentence in the discussion section to emphasise that again, see page 30.

### Comment 12:

Minor comments:

- Wording in the abstract (lines 53-54) and in discussion: the authors state that ‘one quarter of Yemen districts had severe or moderate *A. lumbricoides* infections but the wording implies intensity of infection. Also, ‘infection rate’ is used (line 71) which most often refers to incidence rate and not prevalence, which is measured in this study. I suggest modifying the wording to make clear that they are referring to prevalence of infection.

**Response:** We have made the suggested changes in the abstract and the discussion. We have changed ‘severe or moderate *A. lumbricoides* infections’ to ‘severe or moderate levels of *A. lumbricoides* infection’ and ‘infection rate’ to ‘level(s) of infection’. See page 3 and 4

### Comment 13:

- There is inconsistency throughout the manuscript with the use of decimals when referring to prevalence

**Response:** We have standardized that throughout the manuscript

### Comment 14:

- Few typographical errors throughout the manuscript that need correcting.

**Response:** We have reviewed the manuscript and made corrections to typographical errors.

## **Reviewer 2**

### Comment 1:

**Abstract**

= Please for the abstract, consider the submission guideline of PLoS Negl Trop Dis, particularly for the background which I found very long

“The Abstract should be succinct; it must not exceed 250–300 words. Authors should mention the techniques used without going into methodological detail and summarize the most important results with important numerical results given.”

**Response:** We have reviewed the abstract and made corrections to make sure it fits with the guidelines of the journal and reduced to fewer than 300 words.

### Comment 2:

Background

= “Secondarily, the study aimed to provide a broad indication of the impact of large-scale treatment on the distribution of infection.” This secondary objective of this paper seem cumbersome and was not properly assessed in the current paper due to the design of the study then, it should be removed.

**Response:** This is similar to comment 3 from reviewer 1. The main objective of the paper is to describe the nationwide distribution of SCH and STH infection and anaemia prevalence using the results of the 2014 survey. As a secondary objective, we do also reference the earlier, baseline, mapping to give an indication of changes in infection distribution. We have provided more information on the design of the earlier mapping on page 6. We have inserted the following in on page 7 that the methodology for the two surveys was not identical which precludes a direct comparison. However, we feel presenting the data (with these caveats) is still useful.

“…evaluating the progress, the programme had made against SCH compared to the baseline situation and to potentially shift from an objective of control to elimination. Although we note that the baseline and current surveys used different methodologies meaning a direct comparison was not possible.”

### Comment 3:

= L50 – L51 Write “…with 65.1% [xx/333] and 24.4% (xx/333) districts endemic respectively” instead of “…with 65.1% and 24.4% districts endemic respectively” to ease the reading and understanding.

**Response:** This has been changed

### Comment 4:

Introduction
= “Before large-scale control programmes were undertaken in Yemen.” When? Regarding the topic of the manuscript this an important information

**Response: this was in 2008, this was added in page 5.** Also, more information about the background of the program were added to page 6 (highlighted in yellow).

### Comment 5:

Materials and Methods

= L137 – L138: “A countrywide cross-sectional school-based survey was conducted in 2,664 schools in 332 health districts between February to May 2014.” How the impact of PC has been assessed with a cross-sectional study?

**Response**: While we agree with the reviewer that longitudinal surveys are regarded as the gold-standard of assessing impact of an intervention, it is a common practice in applied research and public health to use pre and post cross-sectional studies to assess impact of health program or interventions where it is not logistically feasible to use longitudinal surveys, especially in low-income countries. This is the case here where we are comparing district level estimates of prevalence of infection rather than infection status in individuals. More information on that could be found in these references:

- Craig, P., Dieppe, P., Macintyre, S., Michie, S., Nazareth, I. & Petticrew, M. 2008. Developing and evaluating complex interventions: the new Medical Research Council guidance. *Bmj,* 337**,** a1655.
- Eldredge, L, K., Markham, C. M., Ruiter, R. A., Kok, G. & Parcel, G. S. 2016. *Planning health promotion programs: an intervention mapping approach*, John Wiley & Sons.
- Kreuter, M. W., McQueen, A., Boyum, S. & Fu, Q. 2016. Unmet basic needs and health intervention effectiveness in low-income populations. *Preventive Medicine,* 91**,** 70-75.
- Michie, S., Atkinds, L. & West, R. 2014. *The behaviour change wheel: a guide to designing interventions,* Great Britain, Silverback Publishing.

### Comment 6:

**Study design and population**

= L160 write “The data were” instead of “The data was”

**Response**: Changed, see page 8

### Comment 7:

= L160: what do you mean by “The data was collected by 25 teams, including five parasitological technicians” is each team had five technicians or the five technicians were for the 25 teams. That call for clarification since the quality of the data could be linked to this.

**Response**: We have clarified in the text (page 8). There were 25 teams, each of which contained five parasitological technicians.

### Comment 8:

= L165 “…obtained their informed consent to be screened.” Is it oral or written consent!? And who was giving the consent? Note that children are assenting but could not consent.

**Response**: We have clarified in the text (page 8). Written consent was obtained from school headteachers, and oral assent was received from the children.

### Comment 9:

**Ethical considerations**

= L242: Please my comment above on the consenting process >>written and signed or thumb-printed informed consents were taken from parents or guardians, on behalf of their children

**Response**: As above, we have clarified that written consent was from the school headteachers, and oral assent was received from the children, see page 12.

### Comment 10:

= L245 – L247: I am not sure if that is true based on my comment above

**Response**: As stated above, written consent was from the school headteachers, and oral assent was received from the children, this has been clarified in page 12.

### Comment 11:

Results

= L251 – L252: this is already said in methods and could be removed from the results

**Response:** Removed, thank you for the good observation

### Comment 12:

= L249 – L254: Please, for consistency, provide a subtitle to this part

Response: Done

### Comment 13:

= L256 – L262: “This equates to 145,919 infected pupils and 2,810,518 of the general population warranting MDA for SCH or STH...” This may be included in the discussion and should be removed from here

**Response:** were considered.

### Comment 14:

= Please, revise the title of the figure

For example write “Figure 1. Maps Showing Prevalence of (a) Intestinal (*Schistosoma mansoni*) and (b) Urogenital (*S. haematobium*) schistosomiasis infection in Yemen, 2014” instead of “Figure 1 Point prevalence maps of (a) Intestinal (*Schistosoma mansoni*) and (b) Urogenital (*S. haematobium*) schistosomiasis infection in Yemen, 2014”

= Same for figure 2

**Response:** The headings of Figures 1 and 2 have been changed accordingly.

### Comment 15:

= “**Figure 3: Changes in the endemicity of schistosomiasis in Yemeni districts between**

**2010 and 2014**” Please, nowhere in the methods it has described how the data on this issue have been gather or collected. Please, I will strongly advice to remove this from the manuscript.

**Response:** We have provided more information about the earlier mapping surveys, see page 5 and 6. Although the comparison is not perfect given different mapping methodologies, we do feel it is worthwhile to show the changes from baseline. However, we do see your point here. Therefore, we have moved this figure to be part of the discussion section instead of the results section.

### Comment 16:

**Sample collection and laboratory procedures**

= “We enrolled 489 schoolchildren…..period from February to August 2017” that means seven months in total for the duration of the study. Then, the cross-sectional study the authors are talking about in the “Study design and population” is not true. I thing that this study is a “Longitudinal non-comparative study”

**Response:** Although we do not think this is referring to our study as our study was conducted in 2014. However**,** we are grateful to have the opportunity to clarify here. We feel confident that the study is cross-sectional rather than longitudinal. Although the surveys took several months to complete, this is not unusual for a mapping exercise of this size and complexity. Each area (individual, school, and district) was only visited once during the 2014 survey, so this study is not a longitudinal study.

### Comment 17:

= “All infected…biltricide praziquantel (40 mg/kg body weight).” This part should belong to the ethics

**Response:** We have added this to the ethics section, page 13.

### Comment 18:

= write “Cross-reactivity of the CCA strip test was evaluated by examination of selected groups whom were known positive for common frequent pathogens namely, *Hymenolepis nana*, *Leishmania donovani*, Hepatitis B viru, *Entamoeba histolytica*,

*Giardia lamblia*, *Plasmodium falciparum* and patients with urinary tract

infection.” Otherwise, this is already results you are providing

**Response:** We do not think this is referring to our study. We did not use CCA strips. The quotation above seems to be from another study.

### Comment 19:

= “In addition, 20 healthy blood donors were tested for the CCA urine strip…subjected to CCA urine strip test evaluation.” This has never been raised as an objective of the current study. Please, could you remove this part and remain focus on the main objective.

**Response:** We do not think this is referring to our study. We did not use CCA strips. The quotation above seems to be from another study.

### Comment 20:

= “…then the same forty samples were put in the refrigerator (4 up to 8 ºC) for seven days and then for one year at -20 ºC.” it seems that the study was not taking seven months as stated above but a full year and maybe more. Please note that the reproducibility of scientific work is very important then man should carefully consider that when writing a paper.

**Response:** We do not think this is referring to our study. We did not use CCA strips. The quotation above seems to be from another study.

### Comment 21:

= “These particular situations …..whenever it demanded.” Should belong to the results

### **Response:** We do not think this is referring to our study. The quotation above was not mentioned in any part of our study, it seems to be from another study.

### Comment 22:

**Evaluation of post-treatment efficacy of CCA test strips**

= I know that many authors have the temptation to introduce the results in the description of the methods but that should not be the case. This whole section should be revised to consider this point

**Response:** We do not think this is referring to our study. We did not use CCA strips. It seems to be discussing another study.

### Comment 23:

**Data analysis**

= Please write “Stability of CCA in urine samples was determined by comparing of the test line intensity throughout the storage approaches with the initial band intensity at baseline.

**Response:** We do not think this is referring to our study. We did not use CCA strips. It seems to be discussing another study.

### Comment 24:

**Discussion**

= “Nevertheless, in the current study, although most of the infected subjects (46.3%) had light infections as determined by Kato-Katz method” This has never been mentioned in the results and seems to be a new information

**Response:** We do not think this is referring to our study. The quotation above was not mentioned in any part of our study, it seems to be from another study.

## Added References

Nagi, M.A.M. (‎2005)‎. Evaluation of a programme for control of Schistosoma haematobium infection in Yemen. EMHJ - Eastern Mediterranean Health Journal, 11 (‎5-6)‎, 977-987, 2005 <https://apps.who.int/iris/handle/10665/117028>

World Bank appraisal document 53766-YE, Dec 2010 <https://documents1.worldbank.org/curated/en/283341468340753068/pdf/537660PAD0Revi1rd10IDA1R20111001011.pdf>
