# Supplementary material for: National mapping of schistosomiasis, soil-transmitted helminthiasis and anaemia in Yemen: Towards better national control and elimination
Source: PLoS Negl Trop Dis. 2022 Mar 25;16(3):e0010092. doi: 10.1371/journal.pntd.0010092 (PMC8986123; doi:10.1371/journal.pntd.0010092)
Supplement: Attachment — Submitted filename: Cover Letter_ 1 Dec 2021.docx [file pntd.0010092.s006.docx]

**Responses to Reviewer's Questions**

**National Mapping of Schistosomiasis, Soil-Transmitted Helminthiasis and Anaemia in Yemen: Towards Better National Control and Elimination**

**PLOS Neglected Tropical Diseases: PNTD-D-21-00800**

Dear Editor,

Thank you for the second opportunity to amend the manuscript and to resubmit it to PLOS NTDs. We thank the anonymous reviewers as well for their subsequent review and constructive comments. We have endeavoured to incorporate these suggestions wherever possible, and we feel that these have resulted in a stronger manuscript that better communicates this important area of public health.

Below we provide individual responses to the reviewers’ comments and suggestions and highlight the relevant line numbers from the manuscript.

Thank you for considering this revised version, we look forward for a successful publication with PLOS NTDs.

Dhekra Annuzaili, on behalf of the authors.

**Reviewer #3**

**Comment 1:**
Lines 121-22:  The authors have now provided the years when the 45 million tablets distributed to 13 million people; however, it’s hard to understand how these numbers relate to the number of years of treatment and coverage.

**Response**

Thank you for this important comment. We have moved this point about the first three years of the programme into the discussion section where we have provided more information (lines 362 – 369). This helps explain what has been implemented to reduce the prevalence in 2014 compared with 2010.

We hope this clarifies the issue.

**Comment 2:**
Line 204-205: “The results were then cross-checked before being to the SCI.”   A word is missing here.

**Response**

The verb ‘’sent’’ has been added (line 202). Thanks to the reviewer for this precise note.

**Comment 3:**

In this manuscript, Johari and colleagues report the results of a large-scale mapping exercise for schistosomiasis and soil transmitted helminths that was conducted in 2014. The scale of this survey was impressive – more than 80,000 children tested – makes these data especially noteworthy.  It is a pity that there was such a delay in the reporting of this information and that the war has undermined the ability to provide MDA.

**Response**

Thank you for your comments on the data. We agree that there has been an extended, non-intentional delay in reporting this important information caused by the security situation in Yemen. Although thoughts of publishing the survey were planned early even before its implementation. Meanwhile, the Yemen MOH and the SCI in collaboration with the WHO and the World Bank announced the results of the survey in the WHO-HQ website at the end of 2014. However, the emerging political unrest and the eruption of the Yemen war in early 2015, and the subsequent suspension of project implementation and refocusing of human resources on other health priorities in Yemen delayed its preparation for publication in a peer-reviewed journal.

We believe that the paper still includes information relevant to helminth control in this region and beyond. We also hope that publishing the results of the survey will help to advocate for future funding for implementation of similar activities in Yemen to secure the gains that have already been made against these diseases.

**Comment 4:**

I still have a few concerns about this paper. First, as noted in the discussion, the authors are trying to make the case that the 2014 survey results document “substantial and significant reduction in the burden of both SCH and STH.” Despite some additions to the text, the reader still doesn’t have access to the 2010 survey results or even an adequate description of the methodology. If the comparison of the 2010 and 2014 results is a central conclusion of the paper, more information should be provided about the 2010 survey.

**Response**

Thank you for the opportunity to clarify and to amend the manuscript accordingly. We have provided further caveats that a direct comparison is not possible between the 2010 and 2014 surveys and the reasons for that in the discussion section (lines 345-357).

The results of the 2010 survey and any comparison between 2010 and 2014 have been removed from the abstract and author summary so that they are less prominent.

We have added a caveat to the introduction about comparing 2010 and 2014 on lines 111-118 and 143-144.

We have amended the language so that no ‘significant’ reductions are referred to as these would not be appropriate.

As before, additional information on the methods for the 2010 mapping are provided on lines 345-357.

**Comment 5:**

I don’t understand why this paper is using thresholds for SCH that are different from those used by WHO.  I understand the desire to be more conservative, but the programmatic decision can be made separately from what is reported here.

**Response**

We agree with this comment that the paper should use the WHO cut-off points instead of those used for programmatic decision making. Therefore, we have updated the paper throughout to reflect the WHO standard cut-offs of <10%, ≥10 and <50%, and ≥50% for low, moderate and high-risk categories for SCH, respectively. All figure legends and tables have also been updated to reflect this change. The map legends for SCH and STH were also updated to change the term ‘medium’ to ‘moderate’ risk to standardise its use throughout the paper.

However, please note that with the update to the WHO standard cut-offs, the distribution of districts by risk category for SCH remain the same as before as the highest prevalence for both *S. mansoni* (35.7%) and *S. haematobium* (10.2%) infections do not exceed 40%. Therefore, the proportion of districts in associated tables and maps did not change (Lines 278-279 and 283-285).

**Comment 6:**

Given the scale of the tragedy that has unfolded in Yemen, I think it is important for the paper to refer to this more specifically. Are children in school? Is MDA ongoing anywhere? How has the disruption in services impacted children in terms of their growth and anemia status? Are the “gains” being reversed?

**Response**

Thank you so much for raising these very important and crucial questions. We agree that it is a humanitarian tragedy in Yemen. We have provided information in lines 407-430 in the discussion section that explain the effect of conflict on the related programme infrastructure in Yemen. We also referred to some studies related to the prevalence of anaemia in the country.

**Reviewer #4**

**Comment 1:**

The authors have satisfactorily addressed my original comments in regard to methods with the exception of the use of baseline (please see my comments in the summary/general comments section below).

**Response**

Thank you for the suggestion. We have changed ‘baseline/follow-up survey’ to ‘2010/2014 survey’.

**Comment 2**:

I suggest moving the newly added figures in the discussion section to the supplementary folder. The discussion is not the place for presenting this data visually.

**Response**

We have removed any figures from the discussion section. On reflection, we decided to remove Figure 5 completely from the manuscript.

**Comment 3:**

The authors have done well to address all the reviewer comments so thoroughly. Although I agree with the authors response to my comment (#3) regarding the use of previous mapping data to highlight the progress made, I still disagree with the use of the term 'baseline' for this earlier data. I strongly suggest avoiding the use of baseline and follow up as it suggests a very different type of analysis than what has been presented in this manuscript. Rather, the data could be referred to by the year, e.g., 2010 survey and 2014 survey.

**Response**

This is a reasonable point. The ‘baseline/follow-up survey’ has been changed to ‘2010/2014 survey‘ throughout the paper.

--------------------


Figure Files:
While revising your submission, please upload your figure files to the Preflight Analysis and Conversion Engine (PACE) digital diagnostic tool, [https://pacev2.apexcovantage.com](https://pacev2.apexcovantage.com/). PACE helps ensure that figures meet PLOS requirements. To use PACE, you must first register as a user. Then, login and navigate to the UPLOAD tab, where you will find detailed instructions on how to use the tool. If you encounter any issues or have any questions when using PACE, please email us at [figures@plos.org](mailto:figures@plos.org).
 
Data Requirements:
Please note that, as a condition of publication, PLOS' data policy requires that you make available all data used to draw the conclusions outlined in your manuscript. Data must be deposited in an appropriate repository, included within the body of the manuscript, or uploaded as supporting information. This includes all numerical values that were used to generate graphs, histograms etc.. For an example see here: <http://www.plosbiology.org/article/info%3Adoi%2F10.1371%2Fjournal.pbio.1001908#s5>.
 
Reproducibility:
To enhance the reproducibility of your results, we recommend that you deposit your laboratory protocols in [protocols.io](http://protocols.io/), where a protocol can be assigned its own identifier (DOI) such that it can be cited independently in the future. Additionally, PLOS ONE offers an option to publish peer-reviewed clinical study protocols. Read more information on sharing protocols at <https://plos.org/protocols?utm_medium=editorial-email&utm_source=authorletters&utm_campaign=protocols>

__________________________________________________
In compliance with data protection regulations, you may request that we remove your personal registration details at any time.  (Use the following URL: <https://www.editorialmanager.com/pntd/login.asp?a=r>). Please contact the publication office if you have any questions.
