# Supplementary material for: National mapping of schistosomiasis, soil-transmitted helminthiasis and anaemia in Yemen: Towards better national control and elimination
Source: PLoS Negl Trop Dis. 2022 Mar 25;16(3):e0010092. doi: 10.1371/journal.pntd.0010092 (PMC8986123; doi:10.1371/journal.pntd.0010092)
Supplement: Attachment — Submitted filename: Reviewer Comment (24 6 2021).docx [file pntd.0010092.s003.docx]

Author   : Johari et al.
Title       : National Mapping of Schistosomiasis, Soil-Transmitted Helminthiasis and Anemia in

Yemen: Towards Better National Control and Elimination

Journal    : PLoS Neglected Tropical Diseases

Reference: PNTD-D-21-00800    

**General comment**

Efforts have been made to improve the current manuscript. Authors could trust to consider the comments and suggestions below for improvement.

**Abstract**

**= Please for the abstract, consider the submission guideline of PLoS Negl Trop Dis, particularly for the background which I found very long**

**“**The Abstract should be succinct; it must not exceed 250–300 words. Authors should mention the techniques used without going into methodological detail and summarize the most important results with important numerical results given.**”**

**Background**

= “Secondarily, the study aimed to provide a broad indication of the impact of large-scale treatment on the distribution of infection..” This secondary objective of this paper seem cumbersome and was not properly assessed in the current paper due to the design of the study then, it should be removed.

= L50 – L51 Write “…with 65.1% [xx/333] and 24.4% (xx/333) districts endemic respectively**” instead of “**…with 65.1% and 24.4% districts endemic respectively**” to ease the reading and understanding.**

**Introduction**
= “Before large-scale control programmes were undertaken in Yemen.” When? Regarding the topic of the manuscript this an important information

**Materials and Methods**

**= L137 – L138: “**A countrywide cross-sectional school-based survey was conducted in 2,664 schools in 332 health districts between February to May 2014.**” How the impact of PC has been assessed with a cross-sectional study?**

**Study design and population**

= L160 write “The data were” instead of “The data was”

= L160: what do you mean by “The data was collected by 25 teams, including five parasitological technicians” is each team had five technicians or the five technicians were for the 25 teams. That call for clarification since the quality of the data could be linked to this.

= L165 “…obtained their informed consent to be screened.” Is it oral or written consent!? And who was giving the consent? Note that children are assenting but could not consent.

**Ethical considerations**

**= L242: Please my comment above on the consenting process**

**= L245 – L247: I am not sure if that is true based on my comment above**

**Results**

**= L251 – L252: this is already said in methods and could be removed from the results**

**= L249 – L254: Please, for consistency, provide a subtitle to this part**

**= L256 – L262: “**This equates to 145,919 infected pupils and 2,810,518 of the general population warranting MDA for SCH or STH...” This may be included in the discussion and should be removed from here

= Please, revise the title of the figure

For example write “**Figure 1. Maps Showing Prevalence of (a) Intestinal (*Schistosoma mansoni*) and (b) Urogenital (*S. haematobium*) schistosomiasis infection in Yemen, 2014**” instead of “**Figure 1 Point prevalence maps of (a) Intestinal (*Schistosoma mansoni*) and (b) Urogenital (*S. haematobium*) schistosomiasis infection in Yemen, 2014**”

= Same for figure 2

= “**Figure 3: Changes in the endemicity of schistosomiasis in Yemeni districts between**

**2010 and 2014**” Please, nowhere in the methods it has described how the data on this issue have been gather or collected. Please, I will strongly advice to remove this from the manuscript.

.

**Sample collection and laboratory procedures**

= “We enrolled 489 schoolchildren…..period from February to August 2017” that means seven months in total for the duration of the study. Then, the cross-sectional study the authors are talking about in the “Study design and population” is not true. I thing that this study is a “Longitudinal non-comparative study”

= “All infected…biltricide praziquantel (40 mg/kg body weight).” This part should belong to the ethics

= write “Cross-reactivity of the CCA strip test was evaluated by examination of selected groups whom were known positive for common frequent pathogens namely, *Hymenolepis nana*, *Leishmania donovani*, Hepatitis B viru, *Entamoeba histolytica*,

*Giardia lamblia*, *Plasmodium falciparum* and patients with urinary tract

infection.” Otherwise, this is already results you are providing

= “In addition 20 healthy blood donors were tested for the CCA urine strip…subjected to CCA urine strip test evaluation.” This has never been raise as an objective of the current study. Please, could you remove this part and remain focus on the main objective.

= “…then the same forty samples were put in the refrigerator (4 up to 8 ºC) for seven days and then for one year at -20 ºC.” it seems that the study was not taking seven months as stated above but a full years and maybe more. Please note that the reproducibility of scientific work is very important then man should carefully consider that when writing a paper.

= “These particular situations …..whenever it demanded.” Should belong to the results

**Evaluation of post-treatment efficacy of CCA test strips**

= I know that many authors have the temptation to introduce the results in the description of the methods but that should not be the case. This whole section should be revised to consider this point

**Data analysis**

= Please write “Stability of CCA in urine samples was determined by comparing of the test line intensity throughout the storage approaches with the initial band intensity at baseline.

**Discussion**

= “Nevertheless, in the current study, although most of the infected subjects (46.3%) had light infections as determined by Kato-Katz method” This has never been mentioned in the results and seems to be a new information
